# Caring Ability and Professional Values of Polish Nursing Students—A Cross-Sectional Study

**DOI:** 10.3390/ijerph191811308

**Published:** 2022-09-08

**Authors:** Michał Machul, Magdalena Dziurka, Agnieszka Gniadek, Joanna Gotlib, Aleksandra Gutysz-Wojnicka, Michał Kotowski, Dorota Kozieł, Kamila Krasucka, Anna Obuchowska, Patrycja Ozdoba, Mariusz Panczyk, Aleksandra Pydyś, Izabella Uchmanowicz, Beata Dobrowolska

**Affiliations:** 1 Department of Holistic Care and Nursing Management, Faculty of Health Sciences, Medical University of Lublin, 20-081 Lublin, Poland; 2 Students’ Scientific Association at the Department of Holistic Care and Nursing Management, Medical University of Lublin, 20-081 Lublin, Poland; 3 Institute of Nursing and Midwifery, Faculty of Health Sciences, Jagiellonian University Medical College, 31-501 Krakow, Poland; 4 Department of Education and Health Sciences Research, Medical University of Warsaw, 02-091 Warsaw, Poland; 5 Department of Nursing, School of Health Sciences, Collegium Medicum, University of Warmia and Mazury in Olsztyn, 10-561 Olsztyn, Poland; 6 Collegium Medicum, Jan Kochanowski University of Kielce, 25-369 Kielce, Poland; 7 Faculty of Health Sciences, Wroclaw Medical University, 50-141 Wroclaw, Poland

**Keywords:** caring ability, nursing values, nursing students, nursing, education

## Abstract

Caring ability and professional values developed and shaped during nursing university studies are often recognised as fundamental components of education and professional nursing attitudes. The aim of this study was to analyse the relationship between caring ability and professional values among nursing students and their correlation with selected sociodemographic variables. A cross-sectional study was conducted among a convenience sample of 379 nursing students. During the research, the Polish versions of the Caring Ability Inventory and the Professional Values Scale were used. The overall result in the Professional Values Scale was 108.78 (SD = 16.17)—which is considered average, and in the Caring Ability Inventory 189.55 (SD = 18.77)—which is considered low. Age correlated negatively with the professional values of students in total and in the subscale “care”; in contrast, “gender”, “place of residence” and “financial situation” did not show any correlation with the level of students’ professional values and caring ability. The professional values and caring abilities of nursing students depended on the year and mode of study and the type of university. The results of the study revealed that the caring ability and professional values of nursing students undergo changes during their education.

## 1. Introduction

One of nurses’ basic traits, which should be shaped and developed in the course of their training, is the ability to provide care to patients (*caring ability*). Nkongho [1] defines care as the ability of a person to demonstrate their knowledge, skills and humanistic attitude while performing professional duties within a patient/client relation. This research presents *caring* as the foundation of the nursing profession [2], an ethical value [3] or an ingredient of high-quality patient care [4], which can be acquired through the development of students’ nursing competences during their university education [5]. This paper adopts the definition proposed by Watson, which stipulates that caring is a moral concept of nursing, demonstrated in the form of the specific behaviours of a nurse with a view to promoting and protecting human health [6].

The Theory of Human Caring proposed by Jean Watson [7] focuses on the humanistic approach to patient care. The patient is presented as a socio-cultural, biopsychosocial and spiritual individual. This theory comprises 10 Caritas Processes, which are important for fulfilling the goals of nursing, such as authentic presence; supporting the expression of both positive and negative feelings; openness and mindfulness to one’s own spirituality and existentiality; loving-kindness towards oneself and others; satisfying human needs, including the provision of necessary care; strengthening the mind, body and spirit; and creating and developing caring relationships based on trust [7,8].

It follows from the review of literature that applying the Theory of Human Caring may contribute to positive changes in patients with both somatic and behavioural symptoms and to increased job satisfaction among nurses [8]. Care-oriented interventions in nursing practice include, for example, the use of touch, therapeutic communication, music therapy, and the visual arts [8,9]. A systematic review of the literature by Wei et al. [9] demonstrates that the incorporation of caring principles in the nursing training programme effectively strengthens students’ self-confidence, inspires them to adopt a caring approach towards themselves and others, and helps develop teamwork skills, which in the future may prepare students to cope with difficult situations and provide high-quality patient care.

Guardianship constitutes one of the most important professional values for nurses and midwives. Values are important for a human being to survive and function in the surrounding world. They are shaped both by cultural, socio-economic and social conditions, as well as by education and life and professional experience [10,11]. Professional values are the standards of conduct adopted by a given professional groupThe Code of Ethics for Nurses of the International Council of Nurses (ICN) proposes professional values of nurses such as justice, caring, respect, responsiveness, integrity, empathy, compassion and trustworthiness [12]. In Polish cultural conditions, a set of professional values and principles of a nurse is comprised in the Code of Ethics of a Nurse and Midwife of the Republic of Poland. According to this document, nurses are obliged, inter alia, to provide care to a patient and his or her family in line with current medical knowledge, regardless of their background, social standing, political views, religion or any other considerations [13]. The personal values of nursing students shaped during their theoretical and practical training towards professional values help in creating their professional identity [14], thus impacting the choice of actions taken in everyday nursing practice [15]. The standard of nurses’ professional values is related, among others, to job satisfaction, the quality of patient care and decision-making skills [10,16]. For this reason, it is so important that professional values be adequately promoted among nursing students. These include, among others, protecting patient privacy, maintaining safety, being committed to one’s duties, cooperating and acting in a professional manner, observing mutual respect, taking responsibility for the patient and for practice, observing the obligation to pursue lifelong learning and aiding the development of the profession [17,18]. Finally, it should be remembered that according to the concept of Value Based Practice in nursing, in addition to relying on one’s own values as a nurse, it is absolutely necessary to take into account the values of the patient and other individuals involved in the decision-making process [19,20].

Despite the available findings from analyses on caring and related abilities among nurses [1,4] and findings regarding professional values among nurses and nursing students [10,11], no studies were found in the literature review showing the relationship between these two variables among nursing students [15,17].

## 2. Materials and Methods

### 2.1. Aim

To assess the level of professional nursing values and caring ability among nursing students, their mutual relationships, and their correlation with selected sociodemographic variables.

### 2.2. Study Design

A cross-sectional study among 379 nursing students was carried out from May 2021 to July 2021 in Poland. The study was conducted in line with the Strengthening the Reporting of Observational Studies in Epidemiology (STROBE) guidelines [21].

### 2.3. Study Participants and Settings

A convenience sampling method was used to select the study group. The criteria for inclusion in the study were as follows: (1) the status of a student in the field of nursing at the 3rd year of bachelor’s studies—first cycle studies or the 1st/2nd year of master’s studies—second cycle studies (following a series of practical classes in clinical conditions); (2) informed and voluntarily expressed consent to participate in the study; and (3) access to the Internet. It is worth underlining that students from both levels of nursing studies were chosen to participate in a survey, as our intention was to analyse possible changes in caring ability and professional values. Some second-cycle students are already working as nurses and studying at the same time.

### 2.4. Research Instruments

Three research tools were used.

(1). The Polish version of the Professional Values Scale of Nurses (NPVS-3) was used to evaluate the professional values of nursing students. Originally, NPVS-3 [22] was devised by Weis & Schank in 2017 on the basis of the Nursing Professional Values Scale (NPVS), a tool derived from the Code of the American Nursing Association [23]. The scale consists of 28 items and is divided into three subscales (caring, activism and professionalism). The subscale “caring” refers to patient engagement (individual or community-, group-, family- or population-based); the subscale “activism” focuses on those aspects of the profession that nurses can use to promote health diplomacy or have an impact on health policies, and to preserve the cohesion of their profession. Finally, the subscale “professionalism” pertains to the responsibility for the work environment and practice, as well as nurses’ professional and personal development and authority. The respondents answered the questions using a 5-point Likert scale (from 1—not important to 5—very important). The possible score range was 28–140. The higher the score, the stronger the nurse’s focus on professional values [22]. The original NPVS-3 is characterised by a high Cronbach’s alpha coefficient of 0.94. The Cronbach’s alpha coefficient for the Polish version of the scale was 0.95.
(2). The caring ability of the students was assessed using the Polish version of the Caring Ability Inventory (CAI). The CAI scale [1,24] was created by Nkongho (1990) on the basis of four theoretical assumptions of care: care is multidimensional with cognitive elements and is associated with attitudes, the potential of care is present in all people, care can be learned and care is measurable. The scale consists of 37 questions and three subscales: “knowledge” (14 items), “courage” (13 items), and “patience” (10 items). The answers are provided on a 7-point Likert scale ranging from 1 (strongly disagree) to 7 (strongly agree). The possible score to be obtained ranges from 37 to 259. According to the author of the instrument, scores below 203.1 are considered low, scores 203.1–220.3 are considered medium and scores above 220.3 are considered high [1]. However, we should consider that the Polish version of the CAI consists of 36 questions, and the possible score to be obtained ranges from 36 to 252. The reliability of the CAI tool was measured by Cronbach’s alpha, which ranged from 0.71 to 0.84 [24]. Cronbach’s alpha for the Polish version of the scale was 0.71.
(3). The respondents were also asked to fill in a questionnaire prepared by the authors to collect basic sociodemographic data (age, gender, place of residence, civil status, financial situation) and variables related to their professional training (year of university education, status and type of university, mode of study, professional work).

### 2.5. Data Collection Process

The study was conducted from May 2021 to July 2021 in Poland. Due to restrictions on direct contact during the pandemic, the questionnaire was prepared for online use. The respondents were enrolled through invitations posted on blogs, discussion forums and social network sites devoted to health sciences and nursing. In addition, invitation e-mails were sent to major centres offering education in the field of nursing in northern, central, southern and western regions of Poland. Potential participants were asked to click on the link to the research materials. The message included an invitation to the study, the purpose and course of the study, contact details for any questions about the study, a statement of informed and voluntary consent to participate and questions from three research tools. The respondent could only take part in the study once. The questionnaire was delivered to 877 respondents, with 379 questionnaires (43.2%) correctly filled in and returned. When collecting data using an online questionnaire, the recommendations proposed by Gupta [25] were followed to maintain the reliability of the results. The online survey was formatted using Survio software, which meets ISO 27001, the international standard for information security requirements, and is fully compliant with the processing of personal details pursuant to the German personal data protection act (the strictest act of law of this type in the European Union). Each survey was protected by an international safety certificate with extended validation (Organization Validated SSL Certificate).

### 2.6. Statistical Analysis

The statistical analysis was performed using the IBM SPSS Statistics (version 25) package software. Descriptive methods were used during the statistical analysis. The distribution frequency of the variables was estimated as standard deviation and mean percentages. The assessment covered correlations between the CAI and NPVS-3 scales and selected sociodemographic and educational variables of students (Pearson’s correlation, Kruskal-Wallis test, Mann-Whitney U test, Spearman’s rho). The *p* values obtained from multiple tests were adjusted using the Bonferroni method.

### 2.7. Ethical Issues

Participation in the study was voluntary and anonymous. Before entering the study, the respondents had been informed about the purpose, course and essence of the study and the possibility of withdrawing from the study at any time. The research was carried out after obtaining the consent of the Bioethics Committee at the Medical University of Lublin (ref. no.: KE-0254/289/2020).

## 3. Results

### 3.1. Study Participants

In the study, 379 nursing students participated. The majority of the respondents were women (95.3%), with an average age of 38.4 years (SD = 11.34). More than half of the students lived in urban areas (64.9%), with almost every second of them unmarried (41.4%). The vast majority of students (78.4%) perceived their financial situation as rather good. Detailed characteristics of the respondents are presented in Table 1.

### 3.2. The Level of Professional Nursing Values, Caring Ability, and the Relationship between Them among Nursing Students

The overall result in the Professional Values Scale was 108.78 (SD = 16.17)—which is considered average, and in the Caring Ability Inventory 189.55 (SD = 18.77)—which is considered low. The highest score was observed in the “activism” subscale in NVPS-3 and in the “knowledge” subscale in CAI. The exact results for each subscale are presented in Table 2.

The study revealed a statistically significant correlation between the examined CAI and NPVS-3 scales and all their subscales (*p* < 0.001), (Table 3).

### 3.3. Level of Professional Nursing Values, Caring Ability, and Sociodemographic Characteristics of Nursing Students

We found a statistically significant negative Pearson’s correlation between age and the overall score of nursing professional values (*r =* −0.124; *p* = 0.016) and the “caring” subscale (*r =* −0.165; *p* = 0.001). A positive relationship was also found between age and the caring ability of students in the “knowledge” subscale (*r =* 0.155; *p* = 0.003) (Table 4).

A significant relationship was found between the year of study and the overall CAI result (H = 6.867; *p* = 0.032) and the “knowledge” (H = 7.754; *p* = 0.021) and “courage” CAI subscales (H = 7.905; *p* = 0.019). We also observed a significant relationship between the year of study and NPVS-3 in the “care” subscale (H = 9.449; *p* = 0.009). The lowest NPVS-3 score was obtained by 2nd year students of the MA programme, while the highest was obtained by 3rd year students of the BA programme. At the same time, the highest overall score in CAI can be observed in the respondents from the 1st year of the MA programme, while the lowest in the 3rd year of the BA programme. The CAI “knowledge” subscale exhibited a significant relationship (H = 8.653; *p* = 0.013) with the type of school in which the students had received their education (Table 5). In the case of statistical significance, pairwise comparisons were made using post hoc tests (Supplementary Table S1).

The study also showed a negative correlation between the type of university (public, non-public) and the “care” subscale on the NPVS-3 scale (Z = −2.418; *p* = 0.016). Moreover, the study mode correlated negatively with the overall score on the NPVS-3 scale (Z = −2.098; *p* = 0.036) and its “care” subscale (Z = −2.532; *p* = 0.011). On the CAI scale, the negative correlation with the mode of education occurred only in the “knowledge” subscale (Z = −3.289; *p* = 0.001) (Table 6). The respondents from public universities in full-time programmes received higher overall results on the NPVS-3 scale and lower on the CAI scale, as compared to the respondents from non-public universities and part-time programmes. In addition, the respondents studying at public universities in full-time programmes obtained higher scores on the “patience” subscale on the CAI scale than students from non-public universities and part-time programmes.

The study also showed a statistically significant correlation between the NPVS-3 “care” subscale and the respondents’ work (H = 7.998, *p* = 0.018). The respondents who did not work as nurses demonstrated the highest level of professional values in the “care” and “professionalism” subscales and (in total) in the NPVS-3. In turn, non-working respondents received the highest results in the “activism” subscale in the NPVS-3 (Table 7).

Table 7 also shows the absence of a statistically significant correlation between the respondents’ care abilities and their work. Students who worked in a position other than a nurse showed the highest level of caring ability (M = 191.76; SD = 18.53); lower scores were obtained by students working as nurses (M = 190.10; SD = 18.61) and non-working respondents (M = 187.90; SD = 19.15). However, people working in the nursing profession obtained the highest ‘caring ability’ score in the “knowledge” subscale (M = 69.79; SD = 8.50) and the lowest in the “patience” subscale (M = 63.05; SD = 6.16). The lowest score for caring ability in the “courage” subscale was demonstrated by non-working people (M = 55.70; SD = 9.79).

The study did not show any statistically significant relationship between variables such as place of residence, gender and financial situation with the students’ caring abilities (CAI) and their professional values (NPVS-3).

## 4. Discussion

The aim of the study was to assess the relationship between professional values and the caring ability of nursing students and their correlation with selected sociodemographic variables. Backed by the research tools used in the research, it was the first study of this type in Poland. Additionally, the authors did not find in international publications any mentions of research on the relationship between professional nursing values and caring ability among nursing students.

The overall NPVS-3 score shows that students present an average focus on professional values. Similar results with regard to professional values were obtained by Paşalak et al. [26], where the score was 105.8 (SD = 16.0) among nursing students from Tanzania and 107.9 (SD = 8.4) among those from Spain. In this study, Turkish students obtained higher scores in the professional values (113.5, SD = 12.8) category compared to their peers from Spain and Tanzania. While this result is also higher than that obtained by Polish students, it should be noted that in the Paşalak study, the average age of the surveyed Turkish students was 21.5 (SD = 1.6), while the average age of Polish students was 38.4 (SD = 11.34). At this point, it should also be noted that the average age among nurses in Poland in 2022 was 53.7 [27,28], and as a result of the recent changes in pre-graduate education and legal regulations introducing a wage system dependent on education, a significant proportion of nurses with many years of experience in Poland have undertaken second-degree studies. Significantly higher results than in the case of Polish students were obtained by their colleagues from India with a minimum of one year of professional nursing experience (121.07, SD = 15.32) [29]. The study in India was conducted on a similar age group (31.2 SD = 7.06)—the high average score suggests that Indian nurses exhibit a strong focus on professional values. It can be assumed that the differences in these results may result from various culturally conditioned attitudes and beliefs, which is why future research focusing on the impact of culture on the professional values of nurses is advisable.

The overall score of the CAI indicates that students present with low caring abilities. Similar results were obtained for undergraduate students from China [30,31], and slightly higher scores were obtained for students from America [31]. Professional values and caring abilities may therefore depend on the geographical region in which nursing students receive education.

Among the professional values of nursing students in the “care” subscale, the next items are “activism” and “professionalism”, respectively. Similar results were obtained by other researchers [32,33]. Care is focused on the involvement of nursing staff in the provision of assistance—to individual patients, their families, or the local community [22]. This may suggest that nursing students treat care as one of the fundamental values of helping patients. Based on the questions in the “caring” subscale in NPVS-3 [22], it can be concluded that important issues for nursing students also include the protection of the statutory and moral rights of the patient to care regardless of their religion, nationality and social or financial situation, as well as of their privacy and the need to ensure the confidentiality of data and respect their dignity and values. The students also seemed to find it important to accept responsibility for the activities performed by nurses and to follow the principles of loyalty and respect for others. Activism, on the other hand, can be perceived as a less significant issue than other values related to nursing, as the dimensions mostly focused on in nursing education programmes are care and professionalism [34]. Activism is the responsibility of nursing staff, which involves influencing decisions made between the local and global levels, engaging in collegial activities, providing support and cooperating to ensure professional satisfaction and promoting better access to health care and its results [22,34]. Professionalism reflects the nurse’s sense of responsibility for the work environment, personal and professional development aimed at keeping knowledge and skills up to date, having authority among colleagues, recognising boundaries and observing practical standards of the nursing profession [22]. A viable solution proposed by Poreddi et al. [29] would be to use the role-modelling method in the education of future generations of nurses. Regarding the scores of the scale concerning caring ability, “patience” comes first and is followed by “knowledge” and “courage”. The “knowledge” subscale shows the degree of perception of oneself and the person who is under the nurse’s care; “courage” can be seen as the ability to cope with sudden and unknown situations in the care process; and “patience” is related to acceptance and perseverance in professional practice [1]. Similar results were obtained by researchers from China [30], where the vast majority of respondents were women who displayed greater patience than courage in patient care, as this depends on how much professional experience has been gained [30,34].

Interestingly, in our study, age correlated negatively with the professional values of students in general and in the “care” subscale. This may be due to factors such as clinical experience or the burden of both educational and personal obligations [35]. Therefore, educational measures should be taken to raise students’ awareness of training and the development of professional values, paying attention to the educational methods used in nursing programmes as part of the university curriculum. As stated by Ma et al. [36], the teaching of humanities subjects during nursing education is often theoretical in nature, neglects practice and lacks variety and flexibility. For this reason, it may be important to utilise educational methods, such as role-playing or improvisation, to improve caring abilities and develop professional values.

Furthermore, in our study, demographic variables such as gender, place of residence or financial situation were not related to professional values and caring ability. Very interesting results were obtained by researchers from Spain [37]—they focused on the perspective of professional values through the lens of gender and clinical experience. More often than not, women were characterised by a stronger commitment to professional values than men who studied nursing or worked as nurses. These deliberations can be extended to include qualitative research that should offer an in-depth understanding of the determinants of professional values and caring abilities and an analysis of the current situation, with a view to planning adequate measures that will need to be implemented.

The manifested professional values as well as the caring ability of the nursing students taking part in our study depend on a number of factors, e.g., year of study, type of university or mode of study. Respondents in the 2nd year of the MA programme received the lowest score on the professional values scale, while 3rd year undergraduate students received the highest score. A systematic review of the literature by Parandeh et al. [38] showed that education is an important element in the development of professional values among nursing students. In contrast, year 1 and year 2 Master’s students were characterised by a statistically significant, higher caring ability score than year 3 undergraduate (BA) students. The relationship between the year of study and the overall CAI score, as well as its “knowledge” and “courage” subscales, can be interpreted as the natural consequence of the teaching process, where the student, by acquiring both theoretical and practical knowledge, feels more confident in performing specialised procedures and providing patient care. Experience is a key factor in making caring skills and professional values present among nursing staff, as it allows for the development of a patient-centred caring relationship [39]. During clinical classes, students often experience internal conflicts, which makes them more empathetic and caring towards their patients [31]. Furthermore, increased student–patient contact creates problems in meeting patients’ needs and expectations, which can cause students to become discouraged and less confident in their care skills [31]. In addition, people who knowingly choose their field of study develop a higher standard of professional values during their education and exhibit a greater willingness to provide care to patients [40]. The literature also suggests that nursing students are exposed to intensive clinical placements that make them change their perspectives on their professional identity, affecting their perceptions of the nursing profession [41]. As Ferri et al. [42] study results show, during the three years of an Italian Nursing Course in a specific module, “Principles and techniques of the care relationship”, students developed technical and rational components of caring behaviours. What is more, students perceived a high value of caring behaviour in the dimension: “identifying with patient and being emphatic” or “taking with the patient” [42]. Moreover, among all students taking part in the study, the highest value was observed in the “responding to the individual needs” dimension [42].

As reported in the literature, educational level positively influences professional values [18,43]. The negative correlation between the mode of study and the professional values scale (in particular, the “care” subscale) and the CAI “knowledge” subscale may be due to the fact that people choosing part-time nursing programmes are already working in this field, reconciling their education with work and private lives. Extramural study programmes are condensed to the maximum, which may entail insufficient training in ethical skills in favour of clinical competences and knowledge. Life experience, having one’s own family and better communication skills of older students may also be related to caring ability [4].

The type of work was statistically significantly correlated with the professional values of the respondents in relation to the “Care” subscale. Students who did not work as nurses showed the highest standard of professional values. On the other hand, students who worked in non-nursing positions showed the highest level of caring ability, with the lowest level found in non-working people (the difference was not statistically significant). According to Cheng et al. [31], students with professional experience pay more attention to humanistic care and have a better understanding of professional priorities.

### Study Limitations

There are limitations to this study. The study was conducted online and the data were collected only from 379 nursing students from Poland. In connection with the online questionnaire, the study covered only people with access to the Internet and those who belonged to specific distribution channels. Therefore, the results cannot be generalised. Therefore, conducting further research on the above subject is important to examine in more detail the professional values and caring ability of nursing students. The low proportion of men in the study (4.7%) reflects the number of male nurses in Poland [27,28]. Furthermore, the NPVS-3 and CAI scales measured the importance of professional values and the level of care from the perspective of nurses in a quantitative way; they did not assess the application of these values in nursing practice. Future research should focus on qualitative or mixed-study research to understand nurses’ views on ethical and professional values and the ethical challenges they face when integrating these values into professional practice, and to explore whether the presented level of caring values influences the standard and quality of patient care.

## 5. Conclusions

The manifested professional values and caring ability of nursing students depend on a number of factors associated with the mode of study, the year of university education or sociodemographic data such as age and, consequently, the experience gained. Year 1 and year 2 of master’s degree students were characterised by higher caring ability scores than year 3 of bachelor’s degree students. In contrast, 2nd year of master’s degree students achieved the lowest score on the professional values scale, while the highest score was found in 3rd year bachelor’s degree students. The overall scores obtained by students on the NPVS-3 and CAI scales are not satisfactory and indicate the need for reflection regarding the methods of nursing education. Considering nursing professional values, students should be more encouraged to engage in activities for the benefits of the nursing profession or patients. They should have more opportunities to be involved in work in professional organisations or associations. This in turn might influence their professional identity, caring awareness and finally caring ability.

Investigating and assessing the interdependencies between the variables may in the future contribute to improved caring ability and professional values of nursing students and thus, through the introduction of value-based professional education, lead to an increase in the quality of patient care and job satisfaction of nurses.

## Figures and Tables

**Table 1:** Demographic and other selected variables of the nursing students (*n* = 379).

Variables		*n* = 379	
Age		M	SD
		38.4	11.34
Sex		N	%
	Female	361	95.3
	Male	18	4.7
Place of residence	Urban area	246	64.9
	Rural area	133	35.1
Civil status	Single	157	41.4
	Married	131	34.6
	In cohabitation	72	19.0
	Divorced	14	3.7
	Widowed	5	1.3
Self-assessed financial situation	Rather good	297	78.3
	Definitely good	70	18.5
	Rather bad/definitely bad	12	3.2
Year and mode of studies	student of 3rd year bachelor’s studies	138	36.4
	Student of the 1st year of master’s studies	121	31.9
	Student of the 2nd year of master’s studies	120	31.7
University status	Public university	281	74.1
	Non-public university	98	25.9
Type of university	Medical university	166	43.8
	Other than medical	119	31.4
	Higher vocational school	94	24.8
Mode of education	Full-time programme	223	58.8
	Part-time programme	156	41.2
Is the respondent employed	Yes, as a nurse	224	58.9
	Yes, on a casual basis in a profession other than nursing	35	9.2
	No	121	31.9

**Table 2:** Respondents’ overall results on the CAI and NPVS-3 scales.

		M	SD	Cronbach’s Alpha	Score
NVPS-3	Activism	49.34	8.22	0.92	13–65
	Caring	34.17	5.01	0.89	8–40
	Professionalism	25.27	4.46	0.84	7–35
	Total score	**108.78**	16.17	**0.95**	28–140
CAI	Knowledge	69.10	8.80	0.81	13–91
	Courage	56.87	10.48	0.81	12–84
	Patience	63.58	6.18	0.71	11–77
	Total score	**189.55**	18.77	**0.71**	36–252

**Table 3:** Correlations between the CAI and NPVS-3 scales.

	Knowledge	Courage	Patience	Total Score
Activism	0.279 ***	0.208 ***	0.386 ***	0.374 ***
Caring	0.279 ***	0.311 ***	0.497 ***	0.468 ***
Professionalism	0.346 ***	0.202 ***	0.402 ***	0.407 ***
Total score	0.324 ***	0.258 ***	0.461 ***	0.447 ***

*** *p* < 0.001.

**Table 4:** Correlation between respondents’ ages and the NPVS-3 and CAI scales.

	Age	
	r	*p*
Activism	−0.097	0.058
Care	−0.165 **	**0.001**
Professionalism	−0.083	0.107
NPVS-3	−0.124 *	**0.016**
Knowledge	0.155 **	**0.003**
Courage	0.013	0.797
Patience	−0.030	0.565
CAI	0.070	0.172

r—Pearson’s correlation, * *p* < 0.05; ** *p* < 0.01.

**Table 5:** Relationship between the year and place of study and NPVS-3 and CAI.

	Education Level									Type of School								
	Bachelor’s (BA) Studies (3rd Year)		Master’s (MA) Studies (1st Year)		Master’s (MA) Studies (2nd Year)		Statistics		Pairwise Comparison	Medical University		Higher Vocational School		University Other Than Medical		Statistics		Pairwise Comparison
	M	SD	M	SD	M	SD	H	*p*		M	SD	M	SD	M	SD	H	*p*	
Activism	50.07	7.76	49.65	8.25	48.18	8.64	2.465	0.292	-	49.93	7.43	49.62	7.99	48.29	9.35	1.650	0.438	-
Care	34.75	4.57	34.65	5.00	33.03	5.33	**9.449**	**0.009**	1–2 *; 1–3; 2–3 *	34.75	4.40	33.97	5.28	33.53	5.50	2.382	0.304	-
Professionalism	25.67	4.34	25.61	4.05	24.46	4.89	5.625	0.060	-	25.49	4.02	25.43	4.36	24.83	5.08	0.937	0.626	-
NPVS-3	110.49	15.23	109.92	15.57	105.67	17.46	5.509	0.064	-	110.17	14.02	109.01	16.52	106.66	18.45	1.694	0.429	-
Knowledge	67.42	9.04	70.70	8.61	69.41	8.61	**7.754**	**0.021**	1–2; 1–3 *; 2–3	67.77	8.84	70.76	8.69	69.65	8.62	**8.653**	**0.013**	1–2 *; 1–3; 2–3
Courage	55.62	10.10	58.99	10.32	56.18	10.32	**7.905**	**0.019**	1–2; 1–3 *; 2–3	57.22	10.17	56.55	11.17	56.63	10.42	0.161	0.923	-
Patience	63.66	6.10	63.70	5.90	63.36	5.90	0.199	0.905	-	63.80	5.32	63.95	6.28	62.98	7.15	0.189	0.910	-
CAI	186.70	18.84	193.40	18.39	188.94	18.57	**6.867**	**0.032**	-	188.78	18.40	191.26	19.43	189.26	18.82	0.797	0.671	-

* *p* < 0.05, M—Mean, SD—Standard Deviation, H—Kruskal–Wallis.

**Table 6:** Correlation between university type, mode of education, and the NPVS-3 and CAI scales.

	Status of the School						Mode of Education					
	Public University		Non-Public University		Statistics		Full-Time Programme		Part-Time Programme		Statistics	
	M	SD	M	SD	Z	*p*	M	SD	M	SD	Z	*p*
Activism	49.67	7.85	48.40	9.19	−1.189	0.234	49.95	7.91	48.47	8.59	−1.554	0.120
Care	34.56	4.81	33.07	5.40	**−2.418**	**0.016**	34.82	4.47	33.24	5.57	**−2.532**	**0.011**
Professionalism	25.47	4.35	24.68	4.72	−1.447	0.148	25.59	4.26	24.81	4.70	−1.782	0.075
NPVS-3	109.69	15.41	106.15	18.01	−1.776	0.076	110.35	14.87	106.53	17.67	**−2.098**	**0.036**
Knowledge	68.61	9.04	70.49	7.95	−1.909	0.056	67.92	9.10	70.78	8.08	**−3.289**	**0.001**
Courage	56.76	10.53	57.19	10.37	−0.301	0.763	56.62	10.36	57.22	10.67	−0.571	0.568
Patience	63.75	6.01	63.07	6.64	−0.745	0.456	63.74	6.07	63.35	6.34	−0.614	0.539
CAI	189.12	18.99	190.76	18.15	−0.558	0.577	188.28	19.26	191.36	17.95	−1.293	0.196

M—Mean; SD—Standard Deviation; Z—Mann-Whitney U test.

**Table 7:** Correlation between work and NPVS-3 and CAI.

	Yes, in My Profession as a Nurse		Yes, in a Profession Other Than Nursing		Non-Working		Statistics		
	M	SD	M	SD	M	SD	H	*p*	Pairwise Comparison
Activism	48.89	8.55	49.91	7.62	50.01	7.75	0.798	0.671	-
Care	33.55	5.25	35.29	4.43	35.01	4.53	**7.998**	**0.018**	1–2; 1–3 *; 2–3
Professionalism	25.01	4.57	25.79	4.73	25.60	4.16	1.672	0.433	-
NPVS-3	107.45	17.03	111.00	14.95	110.62	14.68	2.391	0.303	-
Knowledge	69.79	8.50	69.06	9.70	67.83	9.01	3.342	0.188	-
Courage	57.25	10.75	58.50	10.91	55.70	9.79	4.033	0.133	-
Patience	63.05	6.16	64.21	5.74	64.37	6.27	4.636	0.098	-
CAI	190.10	18.61	191.76	18.53	187.90	19.15	0.861	0.650	-

* *p* < 0.05, M—Mean, SD—Standard Deviation, H—Kruskal-Wallis.

## Data Availability

The data presented in this study are available on request from the corresponding author.

## Supplementary Materials

The following supporting information can be downloaded at: https://www.mdpi.com/article/10.3390/ijerph191811308/s1, Table S1: Pairwise comparison.
