# Supplementary material for: Caring Ability and Professional Values of Polish Nursing Students—A Cross-Sectional Study
Source: Int J Environ Res Public Health. 2022 Sep 8;19(18):11308. doi: 10.3390/ijerph191811308 (PMC9517121; doi:10.3390/ijerph191811308)
Supplement: Supplementary file 1 [file ijerph-19-11308-s001.zip › ijerph-1870997-supplementary.pdf]

Table S1. Pairwise comparisons.

Sample 1-Sample 2	Test statistic	SE	Standardized test statistic	Significance	Bonferroni-adjusted significance
**Pairwise comparison between the years of study and Care Subscale.**					
2.00-3.00	-36.188	13.628	-2.655	.008	.024
2.00-1.00	37.986	14.067	2.700	.007	.021
3.00-1.00	1.798	13.598	.132	.895	1.000
**Pairwise comparison between the years of study and Knowledge Subscale**					
3.00-2.00	26.991	13.665	1.975	.048	.145
3.00-1.00	36.228	13.634	2.657	.008	.024
2.00-1.00	9.237	14.104	.655	.513	1.000
**Pairwise comparison between the years of study and Courage Subscale**					
3.00-2.00	6.315	13.667	.462	.644	1.000
3.00-1.00	36.396	13.636	2.669	.008	.023
2.00-1.00	30.081	14.106	2.132	0.033	.099
**Pairwise comparison between the type of school and Courage Subscale**					
1.00-3.00	-22.959	13.150	-1.746	.081	.242
1.00-2.00	-40.413	14.131	-2.860	.004	.013
3.00-2.00	15.454	15.107	1.155	0.248	.744
**Pairwise comparison between work and Care Subscale**					
1.00-3.00	-30.776	12.319	-2.498	.012	.037
1.00-2.00	-36.804	20.096	-1.831	.067	.201
3.00-2.00	6.028	21.193	.284	0.776	1.00

Each row tests the null hypothesis that the Sample 1 and Sample 2 distributions are the same.
Asymptotic significance (2-sided tests) are displayed.
The significance level is .050.
a. Significance values have been adjusted by the Bonferroni correction for multiple tests.
